# Extended Outcomes of Complex Coronary Disease in the Drug Eluting Stent Era

**DOI:** 10.4021/cr38w

**Published:** 2011-05-20

**Authors:** Kevin P. Desrosiers, Jeremiah R. Brown, Craig A. Langner, Mandeep S. Sidhu, John F. Robb, Michael J. Hearne, Peter M. Ver Lee, Mirle A. Kellett, Thomas J. Ryan, John R. O’Meara, Harold L. Dauerman, M. Theodore Silver, Craig A. Thompson, David J. Malenka

**Affiliations:** a Section of Cardiology, Department of Medicine, Dartmouth-Hitchcock Medical Center, Lebanon, NH, USA; b The Dartmouth Institute for Health Policy and Clinical Practice, Dartmouth College, Lebanon, NH, USA; c Catholic Medical Center, Manchester, NH, USA; d Eastern Maine Medical Center, Bangor, ME, USA; e Maine Medical Center, Portland, ME, USA; f Fletcher Allen Health Care, Burlington, VT, USA; g Department of Invasive Cardiology and Vascular Medicine, Yale University School of Medicine, New Haven, CT, USA

**Keywords:** Stent, Sirolimus, Paclitaxel, Revascularization, Restenosis

## Abstract

**Background:**

Several randomized trials comparing bare-metal stents to Drug-Eluting Stents (DES) have demonstrated a significant reduction in Target Vessel Revascularization (TVR) and Target Lesion Revascularization (TLR) exists with the use of drug-eluting stents, without compromising survival. These conclusions are based on restricted inclusion criteria for patients and lesion. It is unknown if these results can be generalized to an unselected patient population and more complex disease. The objective of this study was to determine to what extent the availability of DES has impacted survival, TVR, and TLR in a large regional experience without the restriction of on-label indications.

**Methods:**

Patients registered with the Northern New England Cardiovascular Disease Study Group’s PCI registry with single vessel coronary disease undergoing a first PCI were sorted according to the Bare-Metal stent (BMS) era (2001 - 2002) or the Drug-Eluting stent (DES) era (2004 - 2005) based on the time period their first PCI took place. Totally, 6,093 BMS and 5,651 DES patients were identified. Outcomes of survival, TLR and TVR were measured after one year.

**Results:**

After 1 year, survival was comparable, TLR was reduced by 4.9% (absolute) and TVR was reduced by 5.4% (absolute) in the DES era compared to the BMS era. The TLR/TVR differences remained significant after propensity matching in the DES era vs BMS era (Mortality: HR 1.00, 95% CI: 0.83 - 1.28; TLR: HR 0.40, 95% CI 0.32 - 0.46; TVR: HR 0.44, 95% CI 0.38 - 0.51).

**Conclusions:**

In large regional experience with a consecutive series of patients representing the contemporary practice of PCI, including both on and off label use, the frequent use of DES reduces the risk of TVR and TLR without compromising survival.

## Introduction

The introduction of bare metal stents (BMS) to the practice of balloon angioplasty led to reduced rates of restenosis and reduced rates of Target Vessel Revascularization (TVR) and Target Lesion Revascularization (TLR). The introduction of drug eluting stents (DES) was intended to further reduce the risks of restenosis by preventing the proliferation of vascular endothelial cells and smooth muscle. Several randomized trials have been performed to establish efficacy of individual DES, and the pooled data from these studies has also been analyzed [1-6]. The results indicate a reduction in the rates of TVR and TLR compared to the use of BMS with average absolute reductions of 10 - 15%, with no difference in crude or adjusted survival at one year. These results are based on the data from randomized controlled studies with participants who met eligibility criteria, and had on-label indications for coronary artery stent placement. Specifically, these studies included stable patients with single vessel disease, and excluded all Acute Coronary Syndrome patients and patients with a recent history of myocardial infarction (MI). In contemporary practice outside of the confines of randomized studies, stents are placed into a heterogeneous group of patients for both on and off label indications, frequently in patients having acute myocardial infarctions. While prior observational studies have compared real-world patient outcomes between BMS and DES, these studies have been relatively small, or limited to patients with acute myocardial infarction [1, 7]. Hence, the actual benefit of DES with regard to TLR and TVR in this setting is still not fully understood.

The purpose of this study is to determine the benefit of DES era percutaneous coronary intervention (PCI) as compared to BMS era PCI with regard to survival, TLR and TVR in a non-randomized group of patients receiving stents based on contemporary multi-centered regional practice composed of academic and community practices with no protocol-driven restrictions on who received a coronary stent and angiographic or clinical follow-up.

## Methods

### NNECDSG PCI cohort

The Northern New England Cardiovascular Disease Study Group (NNECDSG) was founded in 1987 as a regional voluntary consortium capturing 100% of the coronary revascularizations and/or valve procedures in northern New England including eight medical centers in Vermont, New Hampshire, and Maine. The group consists of clinicians, hospital administrators, and health care research personnel who seek to improve continually the quality, safety, effectiveness, and cost of medical interventions in cardiovascular disease. The PCI and cardiac surgery registries (for coronary artery bypass grafting and valve surgery) have baseline patient and disease characteristics, procedural variables, and in-hospital outcomes. Cardiac procedures (PCI and surgery) conducted within the NNECDSG are added to a relational database to identify repeat revascularization within the region. Survival data was based on the Social Security Death Index which was ascertained at one year of follow up after index coronary angiography. Mortality was determined by a match of the NNE registry to the Social Security Administration Death Master File (SSDMF), US Department of Commerce National Technical Information Service using social security number and date of birth [8, 9]. For patients with partial matches of these variables, a match score was incorporated to account for key-punch errors in social security numbers, date of births or variations of first or last name. High matching scores were considered matches. Patients that were missing matching information were matched according to known identifiers or were considered lost to follow-up [10]. The SSDMF includes all deaths reported to the Social Security Administration occurring in or outside of the United States, with 92% sensitivity for all deaths [9, 11]. The NNECDSG has Institutional Review Board approval for this PCI data collection and analysis at all participating centers.

### Study cohort

The selective use of DES early following FDA approval and their rapid penetration into clinical practice precluded a direct comparison of outcomes for DES vs BMS patients. Instead, we compared outcomes for patients with one vessel CAD undergoing a first revascularization with PCI in 2001 - 2002, when only BMS were available and just prior to FDA approval of the first DES to market in April 2003 (the BMS-Era Cohort) to comparable patients undergoing PCI in 2004 - 2005 when the majority of stented patients received a DES (the DES-Era Cohort). Patients were selected if they had single-vessel coronary artery disease at the time of the first PCI. Patients were then divided into groups based on the time period in which their primary procedure was performed. BMS-Era cohort: Patients whose primary procedure was performed between 2001 and 2002. DMS-Era cohort: patients with a primary procedure between 2004 and 2005. Of 9,190 consecutive patients undergoing their first PCI between 2001 and 2002, 6,093 had single-vessel disease and were defined as the BMS-era cohort. Of 9,656 patients undergoing their first PCI between 2004 and 2005, 5,651 had single vessel disease and were identified as the DES-era cohort.

As the objective of this study was to determine the effect DES availability had on the defined end-points, as opposed to the actual use of the stent, the cohorts were established based on the date of the index PCI irrespective of whether a stent was placed.

### Endpoints

The cohorts were then compared for outcomes of death, incidence of TLR (TLR defined as repeat intervention within the stent or 5 mm proximal or distal to the stent), and TVR (TVR, defined as a repeat intervention within the same vessel, right, left, circumflex or left main coronary artery) at one year.

### Analysis

Survival analysis was performed using Kaplan Meier and log rank survival analysis. Cox’s proportional hazard regression modeling was used to calculate crude and adjusted Hazard Ratios for DES-era versus BMS-era cohorts with 95% confidence intervals. Known predictors of survival and restenosis were used to adjust: age, sex, presence of diabetes mellitus, peripheral vascular disease, COPD, renal failure, cancer, history of MI, CHF, EF, priority, LAD disease, proximal LAD disease, ACC type B2/C lesion and number of stents placed. Patients between the two groups were also propensity matched by blocks using age, sex, presence of diabetes mellitus, peripheral vascular disease, COPD, renal failure, cancer, history of MI, CHF, EF, priority, LAD disease, proximal LAD disease, ACC type B2/C lesion and number of stents placed. A propensity score was generated with variables significantly associated with the exposure of BMS or DES and categorized into propensity-matched blocks using the pscore algorithm in Stata. Cox’s proportional hazard modeling was conducted using the propensity-matched block strata. Analysis was conducted using conditional logistic regression on the propensity-matched blocks.

## Results

There were small differences in demographics and past medical history between cohorts (Table 1). The majority of patients were between ages 50 - 69 years. The DES-era patients were more likely older, female, with more COPD, cancer and were more likely to have undergone PCI within 7 days of an MI. In the DES-era, 81.7% of patients received at least one DES stent and 14.0% received at least one BMS stent (total 95.7%) compared to 92.4% of BMS-era patients receiving at least one stent. The distribution of coronary lesions was similar between the two groups and measured left-ventricular ejection fraction (LVEF) was also similar. Forty-two point four percent (42.4%) of patients in the BMS era vs 52.6% of patients in the DES era had ACC type B2/C lesions which is an off-label indication for stent placement. DES-era patients had slightly more LAD disease, more ACC type B2 and C lesions, and had more emergent procedures, but were less likely to receive 2 or more stents during a procedure. The hospital mortality was similar between the two groups (Table 2).

**Table 1:** Patient Charateristics

Characteristics	BMS-era (n = 6,093)	DES-era (n = 5,651)	P-value
Age (%, yrs)			
< 50	19.5	18	0.02
50 - 59	29	28.4	
60 - 69	24.6	25.9	
70 - 79	20.1	19.6	
> 80	6.8	8.1	
Sex (%)			
Women	33	31.2	0.04
Men	67	68.8	
BSA (%)			
> 1.8	79.4	81.5	0.03
> 1.6 - < 1.8	15.3	14.4	
< 1.6	5.3	4.1	
Comorbidities (%)			
COPD	9.3	11.2	< 0.01
Diabetes	19.7	20.9	0.11
Peripheral Vascular Disease	9.4	9.1	0.58
Renal Failure			
Dialysis	1.9	1.9	0.73
Creatinine	0.7	0.6	
Cancer	0.2	2.1	< 0.01
Cardiac History (%)			
Prior MI			
none	53.4	50.9	< 0.00
< 7 days	38.7	42.4	
8 - 365	6	5	
> 1 year	1.9	1.7	
CHF (%)			
On Admission	3.5	3.6	0.36
Prior to Admission	1.2	1.4	

**Table 2:** Additional Patient Characteristics

Characteristics	BMS-era	DES-era	P-value
Anatomy and Function			
Diseased Vessel (%)			
CX	18.8	17.8	0.02
RCA	39.4	37.8	
LAD	41.8	44.4	
Proximal LAD	20.1	21.7	< 0.01
Lesion Type (%)			
A	15.3	11.4	< 0.00
B1	42.3	36	
B2	28.2	33.4	
C	14.2	19.2	
Ejection Fraction			
< 40	4.8	5.3	0.11
≥ 40	95.2	94.7	
Procedural Priority and Process			
Priority (%)			
Elective	17.4	22.4	< 0.01
Urgent	58.9	48.5	
Emergent	23.7	29.1	
Process (%)			
POBA	7.6	4.3	< 0.01
BMS	92.4	14	
DES	-	81.7	
> 2 Stents	29.6	22.3	< 0.01
In Hospital Outcomes (%)			
Death	0.8	0.9	0.78
Emergency CABG	0.1	0.0	0.31

**Figure 1:**
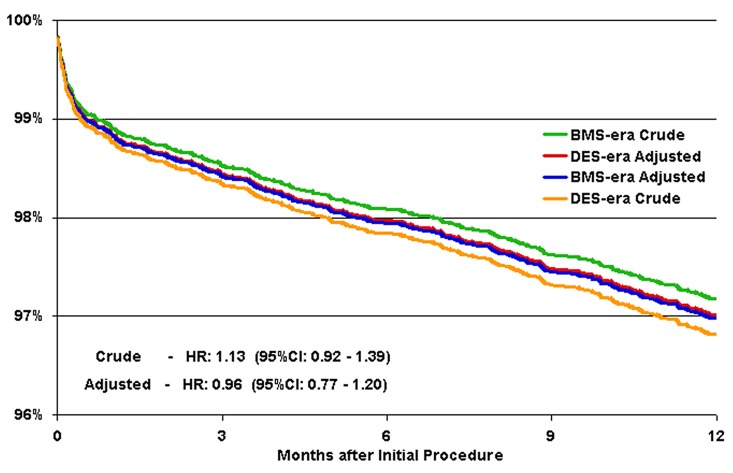
Survival at one-year. Crude and adjusted Kaplan-Meier plot for one-year survival for crude DES era (orange), adjusted DES era (red), crude BMS era (green), and adjusted BMS era (blue).

**Figure 2:**
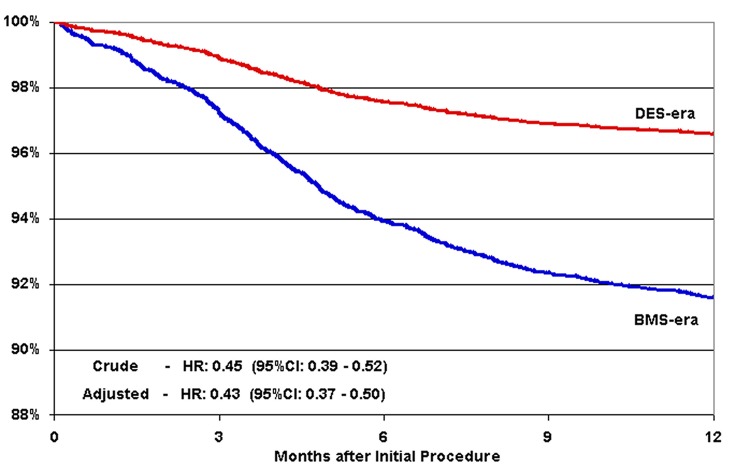
Target lesion revascularization at one-year. Adjusted target lesion revascularization (TLR) time-to-event plot. DES era (red), BMS era (blue).

**Figure 3:**
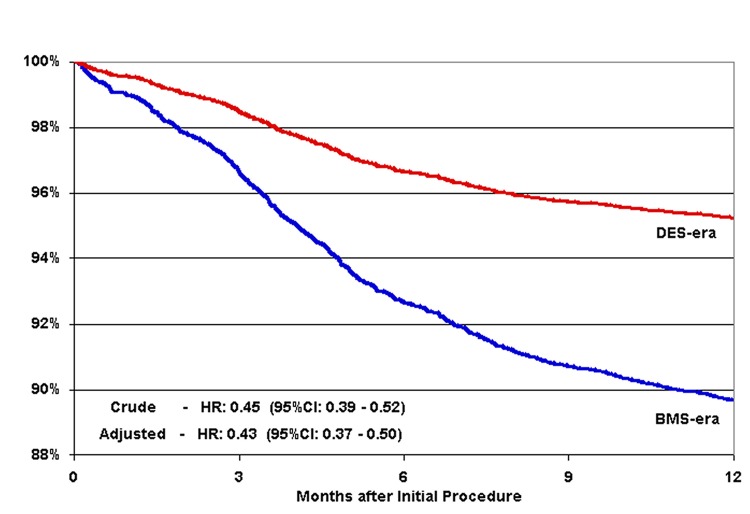
Target vessel revascularization at one-year. Adjusted target vessel revascularization (TVR) time-to-event plot. DES era (red), BMS era (blue).

There was no difference in crude or adjusted survival at one year in the DES-era vs BMS-era cohorts (crude 3.2% vs 2.8% respectively; adjusted HR 0.96, 95% CI 0.77 - 1.20) (Table 3, Fig. 1). Both crude and adjusted TLR were significantly reduced in the DES-era vs BMS-era cohorts (crude 3.4% vs 8.3%; adjusted HR 0.38, 95% CI 0.31 - 0.45) (Table 3, Fig. 2). There were also significant reductions in crude and adjusted TVR in the DES-era vs BMS-era cohorts (crude 4.7% vs 10.1%; adjusted HR 0.43, 95% CI 0.37 - 0.50) (Table 3, Fig. 3). The decreased risk of TLR and TVR for DES-era vs BMS-era cohorts was present across subgroups of patients at increased risk for restenosis (Table 4) including women and those with diabetes mellitus, ACC Type B2 or C lesions, or those receiving ≥ 2 stents. The direction and magnitude of the benefit associated with the DES-cohort was comparable when the analysis was confined to the propensity matched cohort (Mortality: HR 1.00, 95% CI: 0.83 - 1.28; TLR: HR 0.40, 95% CI 0.32 - 0.46; TVR: HR 0.44, 95% CI 0.38 - 0.51) (Table 5).

**Table 3:** Outcomes

Outcome	Crude		Unadjusted Hazard Ratio (95% CI)	Adjusted Hazard Ratio (95% CI)
	BMS-era (%)	DES-era (%)		
Survival	2.8	3.2	1.13 (0.92 - 1.39)	0.96 (0.77 - 1.20)
Target Lesion Revascularization	8.3	3.4	0.40 (0.34 - 0.47)	0.38 (0.31 - 0.45)
Target Vessel Revascularization	10.1	4.7	0.45 (0.39 - 0.52)	0.43 (0.37 - 0.50)

**Table 4:** Outcome in Sub-Groups at Risk for Re-Stenosis

Outcome	DES-era : BMS-era (N : N)	Adjusted Hazard Ratio (95% CI)		
		Survival	Target Lesion Revasc	Target Vessel Revasc
Sex				
Female	1762 : 2007	1.03 (0.74 - 1.44)	0.35 (0.26 - 0.47)	0.42 (0.32 - 0.54)
Male	3889 : 4085	0.92 (0.67 - 1.26)	0.39 (0.31 - 0.48)	0.43 (0.36 - 0.52)
Diabetes				
Yes	1181 : 1200	1.14 (0.74 - 1.75)	0.28 (0.20 - 0.40)	0.33 (0.24 - 0.44)
No	4470 : 4892	0.89 (0.68 - 1.17)	0.41 (0.33 - 0.50)	0.46 (0.39 - 0.55)
BSA				
< 1.8	1034 : 1211	0.71 (0.49 - 1.04)	0.30 (0.20 - 0.44)	0.37 (0.26 - 0.51)
> 1.8	4408 : 4686	1.19 (0.89 - 1.58)	0.39 (0.32 - 0.48)	0.44 (0.37 - 0.53)
ACC Lesion Type				
B2 or C	2908 : 2488	1.01 (0.75 - 1.34)	0.43 (0.34 - 0.54)	0.47 (0.38 - 0.57)
A or B1	2743 : 3604	0.88 (0.61 - 1.27)	0.32 (0.24 - 0.42)	0.39 (0.31 - 0.49)
Number of Stents				
> 2	1330 : 1672	0.85 (0.55 - 1.31)	0.43 (0.32 - 0.58)	0.45 (0.35 - 0.60)
< 2	4321 : 4420	1.00 (0.77 - 1.31)	0.36 (0.29 - 0.44)	0.42 (0.35 - 0.51)

**Table 5:** Outcome in Propensity Matched Patients

	Survival	Target Lesion Revascularization	Target Vessel Revascularization
Adjusted Hazard Ratio (95% CI)	1.0 (0.83 - 1.28)	0.4 (0.32 - 0.46)	0.44 (0.38 - 0.51)

## Discussion

In this large regional experience with a consecutive series of patients representative of the contemporary practice of PCI, the frequent use of DES substantially decreased the risk of both TLR (adjusted HR 0.38) and TVR (adjusted HR 0.43) compared to an era in which use of a BMS was the only stent option. These findings were robust and present in various patient subgroups at increased risk for restenosis, and were achieved without compromising survival. While the current study does not determine whether DES provide a significant reduction in TLR or TVR when compared to BMS, it does provide important insight as to how the wide-spread use of DES during the DES era has affected these outcomes in real-world patients currently receiving stents.

The randomized controlled trials comparing DES to BMS have demonstrated decreased TVR and TLR in populations treated with DES compared to BMS [2, 3]. The SIRIUS Trial reported a reduction in TLR of 12.5% (16.6% in the BMS group vs. 4.1% in the DES group) and a reduction in TVR of 12.4% (21% in the BMS group vs. 8.6% in the DES group) [3]. Similarly the RAVEL Trial showed a reduction in TLR of 15.7% (26% in the BMS group vs. 10.3% in the DES group) [2]. The study by Stone et al, pooling data from four randomized trials found TLR reduction of 15.8% for sirolimus stents vs BMS and 9.9% for paclitaxel eluting stents vs. BMS [5]. Similarly, the TVR was reduced by 15.4% and 7.5% for sirolimus and paclitaxel eluting stents respectively in comparison to BMS [5]. We observed a reduction in TLR of 4.9% and a reduction in TVR of 5.4%. The decrease in magnitude of reduction in TLR vs. TVR compared to the randomized trials may be explained by the elective revascularization of clinically silent lesions that were intervened upon during scheduled follow up angiography in patients enrolled in randomized trials. Alternatively, the patients in this study that would have been excluded from the randomized trials, including those requiring urgent or emergent procedures or those with advanced lesion types may be at higher risk for restenosis despite the placement of a DES. The observed reduction in magnitude may also be explained by the fact that the randomized trials consisted of groups of patients who received exclusively a BMS or a DES, whereas our study had a mixed population in the DES era where 14% of patients received at least one BMS.

Several observational studies have demonstrated a reduction in TLR and TVR at rates similar to those reported in this study [1, 7]. Results of a study performed by Lemos et al comparing “real-world” use of DES vs BMS, compared 450 patients receiving bare metal stents vs. 508 patients receiving drug eluting stents and found a reduction of TVR of 5.8% and a reduction in TLR of 3.8% [1]. These results are consistent with the findings of this current report; however, they differ in subgroup analysis in women and diabetics. This difference may be secondary to higher numbers of patients within these subgroups. Interestingly, the magnitude of the reduction in TLR and TVR was similar between the two studies (TVR reduction 5.8% vs 5.4% in this study), suggesting that the magnitude of reduction in “real-world” patients may be lower than that found in randomized trials.

A recent study by Mauri et al comparing DES vs BMS patients retrospectively in patients with acute myocardial infarction compared 4016 patients receiving a DES with 3201 patients receiving a BMS placed during an acute MI [7]. The investigators found an overall reduction in TVR of 4.9% with the use of DES and a reduction of only 3.8% in patients with ST-segment elevation myocardial infarction (STEMI) at two years. This finding supports the notion that patients receiving a DES in the setting of acute infarction have alternate physiology in comparison to their counterparts with stable coronary artery disease and hence receive reduced benefit from the placement of a DES.

A recent paper by Harjai et al investigated the safety of DES vs BMS in a prospective, observational, single-center, contemporaneous “real-world” study over a 3 to 4 year period [12]. This study compared 1180 BMS patients with 1165 DES patients segregated into groups according to on and off label use. In an analysis of secondary outcomes, the study found a reduction in TVR of 11.4% and 9.5% in the on and off label use of DES respectively. This finding confirms that the magnitude of TVR reduction may be lower than in patients seen in randomized controlled trials. Our study differs from the Harjai study in that our paper examined “eras” as opposed to a contemporaneous group with the hope of eliminating selection bias in the operator’s choice to use a DES vs a BMS. Additonally, our study examined both TLR as well as TVR with respect to DES benefit. Finally, our multi-center study enrolled 5 - 6 fold more patients per group.

A study published by Applegate et al also investigated the three-year safety profile of DES vs BMS in an observational, single-center, “era” based trial [13]. The study compared 1164 patients receiving a BMS in the year prior to the FDA approval of DES (2002 - 2003) with 1285 patients who received a DES in the year after DES was fully available (2004 - 2005). This study found a reduction in TVR of approximately 5% (HR 0.4) at one year, with little discernible benefit at 2 and 3 years. As with the studies noted above, these findings support the concept that the magnitude of the reduction in TVR due to DES is significantly lower than results found in randomized trials. Our paper differs from the Applegate study in that the study was multi-centered and enrolled significantly more patients. Further, patients in the Applegate DES-era were excluded if they received a BMS, whereas they were not in our study. This difference highlights the goal of our paper to evaluate how the wide-spread and often off-label use of DES in the DES-era has impacted TLR and TVR, as opposed to trying to directly compare DES vs. BMS in a non-contemporaneous study with regard to these outcomes.

The similarity among results of these observational studies and our study highlights consistent differences between the groups of patients in the randomized trials, and those who are treated in the “real-world” with off-label use of stents. As noted above, many of the real-world patients would be excluded from the randomized trials due to the urgent or emergent nature of their procedures and their advanced lesion types. Of note, at approximately six thousand patients per cohort, the current study offers nearly ten-fold the number of patients described in previous trials and is not limited to patients with acute myocardial infarctions.

Our study has several limitations including potential historical bias. It is possible that other advances in medical care could have contributed to improved outcomes aside from introduction of DES such as guideline based practice and advances in cardiovascular pharmacology and therapeutics [14]. However, patient characteristics did not change to any great extent over time and we did adjust, using both traditional COX regression and propensity matching, for measured differences in case mix and still found a decreased risk of TVR and TLR in the DES-era. Furthermore, this does not invalidate this study’s conclusion that outcomes are better in the DES era for PCI patients meeting these demographics. We did not have core lab assessment of the coronary anatomy that might have been associated with the risk of restenosis (i.e., lesion length). However, we did control for proxies such as ACC lesion type and the number of stents. To the extent that other aspects of the procedural process (i.e., inflation pressures) improved between the BMS-era and DES-era that could have affected our results. We think this is unlikely because we see the effect with the simplest of lesions, the Type A and B1 lesions with the lowest risk for restenosis. Differences in post-procedure medical management across eras could affect our results though we are unaware of any new guideline recommendations in this time frame for medications that would either decrease the risk of restenosis or improve upon the management of chronic stable angina.

Further studies to determine if a difference actually exists between patients with acute MI and those with stable angina with regard to TLR and TVR after DES would be beneficial. Understanding the pathophysiology of this proposed difference may lead to the development of new stenting technologies to improve benefit in this group.

In conclusion, the “real-world” practice of PCI in the DES-era as compared to the BMS-era is associated with a sustained reduction in TLR and TVR in a broad population including “on label” and complex “off label” patients without compromising patient safety.

## References

[1] Lemos PA, Serruys PW, van Domburg RT, Saia F, Arampatzis CA, Hoye A, Degertekin M (2004). Unrestricted utilization of sirolimus-eluting stents compared with conventional bare stent implantation in the "real world": the Rapamycin-Eluting Stent Evaluated At Rotterdam Cardiology Hospital (RESEARCH) registry. Circulation.

[2] Morice MC, Serruys PW, Sousa JE, Fajadet J, Ban Hayashi E, Perin M, Colombo A (2002). A randomized comparison of a sirolimus-eluting stent with a standard stent for coronary revascularization. N Engl J Med.

[3] Moses JW, Leon MB, Popma JJ, Fitzgerald PJ, Holmes DR, O'Shaughnessy C, Caputo RP (2003). Sirolimus-eluting stents versus standard stents in patients with stenosis in a native coronary artery. N Engl J Med.

[4] Roiron C, Sanchez P, Bouzamondo A, Lechat P, Montalescot G (2006). Drug eluting stents: an updated meta-analysis of randomised controlled trials. Heart.

[5] Stone GW, Moses JW, Ellis SG, Schofer J, Dawkins KD, Morice MC, Colombo A (2007). Safety and efficacy of sirolimus- and paclitaxel-eluting coronary stents. N Engl J Med.

[6] Kirtane AJ, Gupta A, Iyengar S, Moses JW, Leon MB, Applegate R, Brodie B (2009). Safety and efficacy of drug-eluting and bare metal stents: comprehensive meta-analysis of randomized trials and observational studies. Circulation.

[7] Curb JD, Ford CE, Pressel S, Palmer M, Babcock C, Hawkins CM (1985). Ascertainment of vital status through the National Death Index and the Social Security Administration. Am J Epidemiol.

[8] Wentworth DN, Neaton JD, Rasmussen WL (1983). An evaluation of the Social Security Administration master beneficiary record file and the National Death Index in the ascertainment of vital status. Am J Public Health.

[9] Brown JR, Cochran RP, MacKenzie TA, Furnary AP, Kunzelman KS, Ross CS, Langner CW (2008). Long-term survival after cardiac surgery is predicted by estimated glomerular filtration rate. Ann Thorac Surg.

[10] Schisterman EF, Whitcomb BW (2004). Use of the Social Security Administration Death Master File for ascertainment of mortality status. Popul Health Metr.

[11] Mauri L, Silbaugh TS, Garg P, Wolf RE, Zelevinsky K, Lovett A, Varma MR (2008). Drug-eluting or bare-metal stents for acute myocardial infarction. N Engl J Med.

[12] Harjai KJ, Orshaw P, Boura J, Sporn D (2009). Comparison of long-term outcomes of bare metal or drug-eluting stent implantation in standard versus off-label coronary narrowings. Am J Cardiol.

[13] Applegate RJ, Sacrinty MT, Kutcher MA, Santos RM, Gandhi SK, Little WC (2009). 3-year comparison of drug-eluting versus bare-metal stents. JACC Cardiovasc Interv.

[14] Malenka DJ, Kaplan AV, Lucas FL, Sharp SM, Skinner JS (2008). Outcomes following coronary stenting in the era of bare-metal vs the era of drug-eluting stents. JAMA.

